# Misperceived invasion: the Lusitanian slug (*Arion lusitanicus* auct. non-Mabille or *Arion vulgaris* Moquin-Tandon 1855) is native to Central Europe

**DOI:** 10.1111/eva.12177

**Published:** 2014-06-17

**Authors:** Markus Pfenninger, Alexander Weigand, Miklós Bálint, Annette Klussmann-Kolb

**Affiliations:** 1 Biodiversity und Climate Research Centre by Senckenberg Naturforschende Gesellschaft, Goethe-UniversitätFrankfurt, Germany; 2 Institute for Ecology, Evolution and Diversity, J.W. Goethe-UniversitätFrankfurt, Germany

**Keywords:** Bayesian coalescent analyses, climate niche modelling, genetic diversity, phylogeographic model selection, population genetics

## Abstract

The Lusitanian slug, presumed to be native to south-west Europe, was ranked among the 100 worst invading species in Central Europe. However, from the very beginning of its recognition in the presumed invasion area, there was little evidence that the species was actually anthropogenically introduced. We investigated the invasive status of the species by comparing specific predictions on the population genetic structure in the invasion area with the pattern actually found. In a DNA-taxonomy approach, the species could not be found in its presumed native range. Using statistical phylogeographic techniques on a mitochondrial (COI) and nuclear (ZF) marker and species distribution modelling, we could show that the species is with very high probability not an invasor, but native to Central Europe. The study underlines the value of statistical phylogeography in rigorously testing hypotheses on the dynamics of biological invasions.

## Introduction

Since early in (pre)history, mankind has translocated species (Wilson et al. 2009; Jones et al. 2013), but the recent globalization has led to an unprecedented increase in deliberate or unintended introductions of alien species into new areas, often with devastating effects for biodiversity, agriculture and ecosystem services (Simberloff et al. 2013). However, recent studies have shown that some perceived anthropogenic introductions were actually rather natural expansions (Snell et al. 2005; Jesse et al. 2009) or took place in prehistoric times (Jesse et al. 2011). Particularly, in scarcely documented cases or where alternative explanations to an anthropogenic introduction are not *a priori* implausible, careful investigations on the actual status of species perceived as invasive should be conducted. Analyses of the spatial distribution of genetic diversity could be helpful in such cases, as different introduction scenarios can be expected to leave markedly different patterns. In particular, for relatively slowly evolving markers like nuclear or mitochondrial DNA haplotypes, such expectations concerning the level of genetic diversity and their spatial distribution for different introduction and subsequent dispersal scenarios can be formulated and tested.

One such example is the case of the nominal Lusitanian slug *Arion lusitanicus* Mabille 1868. First records of this pest species in Central Europe stem from Switzerland and Southern Germany during the 1950s and early 1960s (see references in Schmid 1970). Since then its recognition and the perception of an invasion has spread throughout Central and Northern Europe. The slug can cause considerable damage on wild and cultivated plants (Proschwitz 1997), but its economic impact has not yet been estimated (Fischer and Reischütz 1998). Occurrence of the species is currently reported from Spain, France, Italy, Switzerland, Austria, the Benelux states, Germany, Czech, Slovakia, Croatia, Slovenia, Poland, Bulgaria, Denmark, Norway, Sweden and Finland on the website of the Delivering Alien Invasive Species Inventories for Europe (DAISIE) website, a web portal on invasive species in Europe supported by the European Commission. The same portal lists the species among the 100 worst invasive species.

However, a careful taxonomic analysis by Castillejo (1997, 1998) confirmed by genetic studies (Quinteiro et al. 2005; Colomba et al. 2007) has shown that *A. lusitanicus* as described by Mabille (1868) is endemic to Portugal and specifically different from the perceived invasor. This created some nomenclatural confusion in the literature. The species is currently correctly referred to as *A. lusitanicus* auct. Non-Mabille (e.g. Kappes et al. 2012), but also as *Arion vulgaris* Moquin-Tandon 1855 (e.g. Pianezzola et al. 2013) or is continuously called *A. lusitanicus* only (e.g. Soroka et al. 2009). This is reflected in uncertainty on the area of origin. Schmid (1970) favours somewhat unspecifically south-west Europe, Chevallier (1981) shows a map with its French distribution and DAISIE gives its native range as spanning north-west Spain, the Atlantic coast of France and Southern England (http://www.europe-aliens.org/speciesFactsheet.do?speciesId=52937).

Overall, there is remarkably little direct or indirect published evidence that the species was actually anthropogenically introduced to Central Europe. Everyone seems to agree that the species was passively distributed with imports of vegetables, salad or the like (Schmid 1970; Fischer and Reischütz 1998). However, the species was never found during controls of imported goods, despite 10 other snail species being found on vegetables, garden soil or potted plants (Fischer and Reischütz 1998). Given the taxonomic confusion, the unclear native range and unknown introduction mechanisms, this species presents a good opportunity to test its status as invasive species with genetic markers.

If an invasive *Arion* species was introduced to Central Europe from an area of origin in Southern England, Western France and Northern Spain since the 1950s, we may formulate some hypotheses on its genetic structure in the invasion area. First, we should find the same lineage that plagues Central Europe in one or more of its purported native ranges. Second, if the invasion took place as reported beginning in the 1950s or even earlier in the twentieth century, we do not expect significant evolutionary divergence (expressed as deep sequence divergence) among the area of origin and the invasion area to have happened since then. Basically, all observed haplotypes should have arisen in the area of origin (Dépraz et al. 2008). Third, if the transportation proceeded primarily with commercially distributed plant material (legumes, fruits, crops, decorative plants, etc.) or the soil associated with them, we may assume that

1. The introduced individuals come from several sites in the area of origin,
2. They were introduced into several sites in the invasion area
3. Further passive dispersal in the invasion area must be invoked, given their poor active dispersal capacity (Grimm and Paill 2001), to achieve the current areawide distribution and
4. The process of introduction continues, because the trade of these possible vector goods among the areas in question was and is intense (http://stats.oecd.org/mei/default.asp?lang=e&subject=12&country=EUU).

We may therefore expect that a substantial fraction of the genetic diversity found in the area of origin is also present in the invasion area. However, the random sampling of transported haplotypes should destroy the expected relation between the phylogenetic age and the frequency of haplotypes in equilibrium populations, where older haplotypes should be more frequent (Donnelly and Tavare 1995). Furthermore, the perhaps not random, but geographically deliberate introduction in addition to further passive dispersal in the invasion area is not in line with the expectation that phylogenetically older haplotypes are more widespread in species that have attained mutation–dispersal–drift equilibrium (Watterson 1985). In other words, we may expect the population structure in the invasion area to conform better to a discrete population model that mirrors the vagaries of the passive transportation mode than to a continuous population model that reflects the continuous active dispersal of a dispersal limited species in its natural range. Fourth, as the rapid invasion of a large area is necessarily associated with a substantial demographic expansion, we may expect such an expansion to have left its traces in the haplotype frequency spectrum and/or the shape of the coalescence structure (Griffiths and Tavare 1999). Explosive growth has been shown to skew expected haplotype frequencies in humans, even if the onset of the expansion started only a few dozen generations ago (Keinan & Clark 2012). Fifth, given the recency of the introduction in evolutionary terms and the expected ongoing geneflow, we can expect the climate niche of the species to be conserved; that is, a climate niche model of the invasion area should comprise the native range and *vice versa*. In addition, the projections of the current climate niche on the conditions of the Last Glacial Maximum (LGM) should coincide with the LGM refugia which should in turn harbour most genetic diversity (Cordellier and Pfenninger 2009).

To investigate the invasion status of the taxon, we sampled large adult *Arion* specimen morphologically resembling *A. lusitanicus* in the presumed areas of origin and substantial parts of the invasion area, applied genetic markers and compared these with published sequences. Furthermore, we analysed the population genetic structure of the presumed invasive taxon and modelled its climatic niche to test the predictions outlined previously.

## Materials & methods

### Sampling and voucher deposition

**Figure 1:**
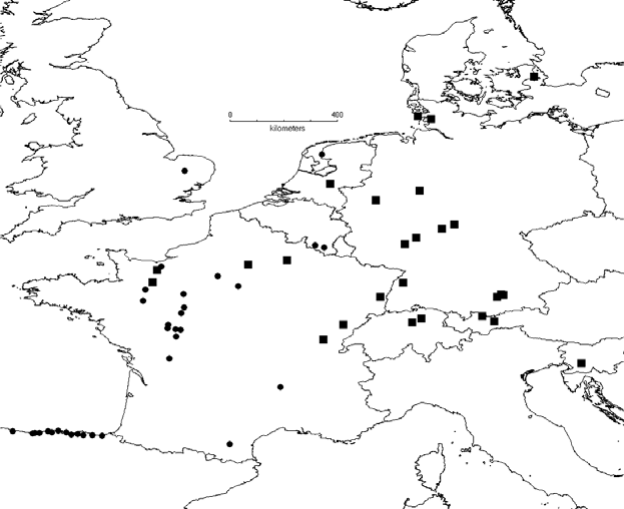
Sampling sites for this study and distribution of Clade 1 (*Arion lusitanicus* auct. non-Mabille or *Arion vulgaris*). Sampling sites are marked with a black dot. If the presumed invasive *Arion* species was found, it was marked with a solid black square.

Specimens were collected in spring of 2010 throughout Western Europe with an emphasis on the presumed area of origin in north-west Spain and south-west France (Fig. 1). We collected adult individuals corresponding to the morphologic description of *A. lusitanicus* as given by the NOBANIS project (Weidema 2006). Whole animals or pieces of the foot were preserved in 80% ethanol. Voucher specimen or tissues were deposited in the Senckenberg Museum für Naturforschung.

### DNA isolation, COI, ND1 and ZF amplification and sequencing

DNA was extracted using the DNeasy Blood and Tissue Kit (Qiagen, Hilden, Germany) according to the recommendations of the manufacturer. PCRs were performed on partial mitochondrial cytochrome c oxidase subunit I (COI) and NADH dehydrogenase subunit 1 (ND1) as well as a nuclear zinc finger-like locus (ZF) with primers and conditions detailed in Appendix S1. The PCR products were directly sequenced in both directions, manually assembled, edited and aligned in MEGA 5 (Tamura et al. 2011). As the nuclear ZF locus is diploid, we checked the trace files for dinucleotide ambiguities. We counted a site as heterozygous single nucleotide polymorphism when the same ambiguity occurred in both forward and reverse sequencing trace file. We inferred the haplotype phases of heterozygous individuals with the coalescent-based Bayesian method PHASE 2.1 (Stephens and Donelly 2003) as implemented in DNAsp 5.10 (Librado and Rozas 2009).

### DNA taxonomy

For the COI data from all *Arion* specimens investigated, we reconstructed a maximum likelihood (ML) phylogeny under the General Time Reversible model with gamma-distributed rates and invariant sites as inferred from model selection. Statistical support for nodes was gained from 500 bootstraps. These analyses were carried out in MEGA 5.0 (Tamura et al. 2011). We considered terminal clades with bootstrap support >90% and at least 3% sequence divergence as operational taxonomic units (OTU, Hebert et al. 2003; Meyer and Paulay 2005). This was not intended as a formal species delimitation approach, even though many if not most so identified OTUs may turn out to be good biological species (Davison et al. 2009). Tentative taxonomic assignments were only derived from 166 published sequences included in the analysis (see list in Appendix S2). Some publications used a mitochondrial ND1 fragment. To make use of this data, we sequenced several individuals from each clade as identified previously for this marker and analysed them together with the 40 published sequences to name yet unidentified clades (see list in Appendix S2). This is possible because COI and ND1 are, as mitochondrial genes, completely linked.

### Phylogeographic analyses

Standard population genetic parameters for the COI and ZF data set were estimated in DnaSP version 5.10 (Rozas et al. 2003). We estimated the average sequence divergence for the COI and the ZF locus among individuals per site in MEGA5 (Tamura et al. 2011) as a measure of genetic diversity. Sequence data for COI and ZF were used to reconstruct statistical parsimony networks in TCS (Clement et al. 2000). Ambiguities were solved as detailed in the study described by Pfenninger and Posada (2002). We used the cladograms to test predictions between phylogenetic age of a haplotype and its distribution and frequency in both data sets (Crandall and Templeton 1993). To this end, we counted the number of mutations connecting a haplotype with the most probable root of the network as inferred by TCS as a measure of relative age. The spread of a haplotype was approximated by the number of sites where it was found. We applied Spearman's rank correlation for a statistical test between the haplotype distribution, frequency and relative haplotype age.

To test whether the phylogeographic structure of the focal species in the presumed invasion area corresponds rather to a discrete (Lemey et al. 2009) or continuous (Lemey et al. 2010) phylogeographic model in BEAST 1.7.5 (Drummond and Rambaut 2007), we applied the respective models for each locus separately and tested their relative support given the data with Bayes factors (Jeffreys 1935). We used a constant population size coalescence model and symmetric trait changes, respectively, a Brownian motion model. All other settings and priors were identical to the EBSP analysis described later. Bayes factors were calculated with the path sampling approach (Ogata 1989). To infer whether the observed pattern of diversification could have arisen after a recent introduction, we compared the unconstrained continuous phylogeographic model for both loci with an identical model where the age of the root was constrained to 100 years, respectively.

### Demographic reconstruction

We used the Extended Bayesian Skyline model EBSP (Heled and Drummond 2008) implemented in BEAST 1.7.5 (Drummond and Rambaut 2007) to infer past demography of the focal species with both COI and ZF simultaneously. As we were dealing with intraspecific data, we enforced a strict molecular clock. Due to lack of a calibration for the taxon, we applied a normally distributed prior with a mean site substitution rate of 2.5 × 10^−7^ per year and a standard deviation of 1 × 10^−7^, truncated to 0 to the COI locus and estimated the relative rate for the ZF locus. The 95% interval of this distribution included thus values between 1% and 9% sequence divergence per million years and was thus conservative regarding estimated values in land snails (Davison et al. 2009).We ran a standard Monte Carlo Markov Chain for 2 × 10^8^ generations, sampling every 10^3^ generations to estimate the posterior distribution of gene genealogies and population parameters under a HKY model with gamma-distributed rates and invariant sites as inferred with the model selection approach implemented in MEGA 5.0 (Tamura et al. 2011). We monitored convergence and effective sampling size in Tracer 1.5. We also applied the haplotype frequency spectrum Tajima's D as implemented in DnaSP version 5.10 (Rozas et al. 2003) to infer past demography.

### Species distribution modelling and genetic diversity

Bioclimatic layers with a resolution of 2.5 arc min for present climatic conditions and for the LGM were downloaded from the public WorldClim database (http://www.worldclim.org, Hijmans et al. 2005). Data for the LGM were drawn from general circulation model (GCM) simulations from the model for interdisciplinary research on climate (MIROC, Hasumi and Emori (2004). The potential present distribution of the species was computed with a maximum entropy approach (Phillips et al. 2004) in Maxent v. 3.3.3 (Phillips and Dudk 2008) based on the presence sites in COI analysis. A general description and evaluation of the method is described by Elith et al. (2011). The models were trained on 75% of the locality information and were tested on the remaining 25%. The predictions were cross-validated in 10 runs. Model performance was evaluated with the area under curve statistics (AUC, Fielding and Bell 1997). The values of the distribution probability maps were transformed into presence/absence values by applying a logistic threshold which maximizes the sensitivity and specificity of the projections.

## Results

### Sampling

We sampled some 300 *Arion* specimen from 60 sites in Central and Western Europe (Fig. 1, Table S1 in Appendix S2). We obtained COI sequences for 285 individuals (GenBank Accession Numbers KJ842822 – KJ843104) and ZF sequences for 87 individuals (GenBank Accession numbers KJ842648 – KJ842821). ND1 sequences were obtained from 39 specimens (GenBank Accession Numbers KJ843105 – KJ843143).

### DNA taxonomy

**Figure 2:**
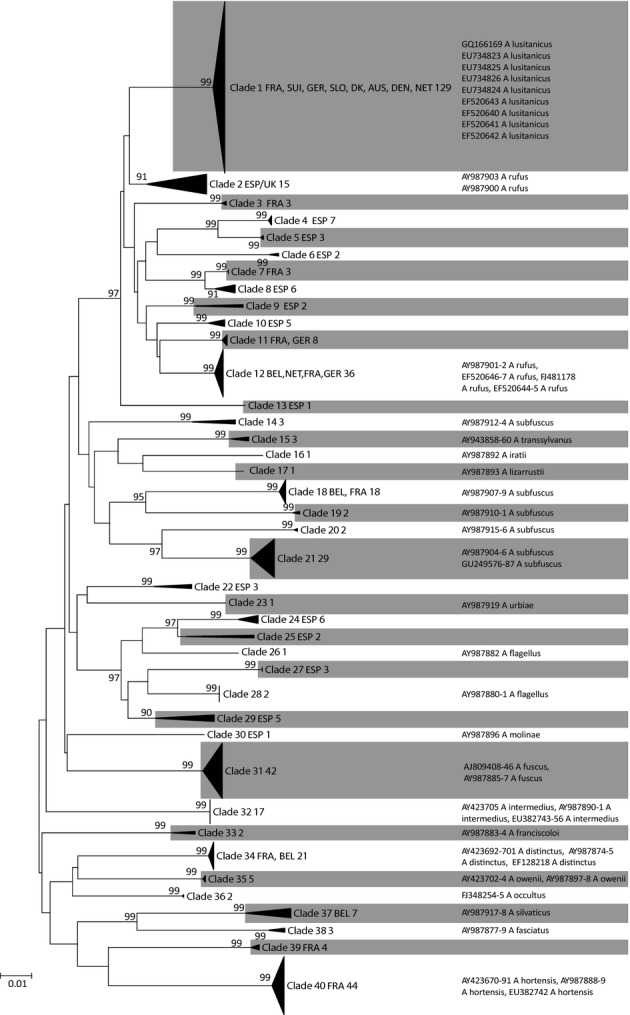
Unrooted maximum likelihood phylogeny of the cytochrome c oxidase subunit I data set. Terminal clades with minimum 90% bootstrap support and at least 3% sequence divergence were collapsed and are depicted as black triangles. Clades were consecutively numbered and abbreviations for the countries were the individuals for this study were found are indicated (ESP, Spain; FRA, France; BEL, Belgium; UK, United Kingdom; DK, Denmark; NET, the Netherlands; SLO, Slovenia; GER, Germany; AUS, Austria; SUI, Switzerland). Number of sequences within each clade is indicated by italicised numbers. If the clades contained published sequences, the GenBank Accession Numbers and their taxonomic designation are given.

Maximum likelihood analysis of COI identified 40 terminal clades with a bootstrap support of 90% or higher and at least 3% average sequence divergence (Fig. 2). Of these, 24 contained a sequence from NCBI with a taxonomic designation. However, the names *A. rufus*, *A. flagellus* and *A. subfuscus* were attributed to at least two, partially highly divergent clades (Fig. 2), respectively. Individuals sampled for this study occurred in 24 different clades, 14 of which could not be attached to a taxonomic name. Published COI and ND1 sequences of two different studies (Quinteiro et al. 2005; Soroka et al. 2009) identified 120 individuals of Clade 1 as the invasive *A. lusitanicus* and thus as the target of this study. The clade was found in Northern and Eastern France, The Benelux states, Germany, Switzerland, Austria, Slovenia and Denmark but not in the presumed native area. The use of ND1 did not increase the taxonomic resolution but confirmed that the individuals of the focal species sampled in Central Europe do not belong to the topotypic *A. lusitanicus* from Portugal (Figure S1).

Of the 24 clades sampled in this study, 10 occurred only at a single sampling site, 11 at 25 sites and three at eight, 10 and 30 sites (Table S1 in Appendix S2). At 40 of the 60 sites sampled, a single clade was found, 11 harboured two, seven sites three clades and at two places, four clades were detected (Table S1 in Appendix S2). All clades showed a more or less restricted and geographically coherent distribution (Figure S2).

### Population genetic analyses and phylogeographic structure

The 120 individuals identified as the focal species showed considerable genetic variation at both the COI (30 haplotypes, 20 polymorphic sites, nucleotide diversity = 0.0056, haplotype diversity 0.753) and the subset of 87 individuals for the ZF locus (22 haplotypes, 28 polymorphic sites, nucleotide diversity = 0.0032, haplotype diversity = 0.573, 43% of the individuals were heterozygous).

**Figure 3:**
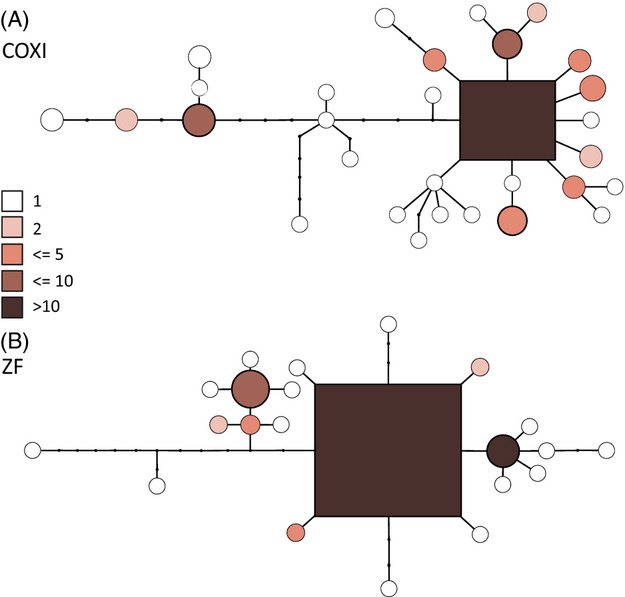
Statistical parsimony networks of cytochrome c oxidase subunit I (COI) and ZF haplotypes of Clade 1. Haplotypes are symbolized by circles or a square if it was the root. The connections between symbols represent base substitutions. Small dots indicate haplotypes that are either extinct or not sampled. The area of the symbols is roughly proportional to their frequency. The colour shading indicates the number of sites where this haplotype was found, with darker shades indicating more sampling sites (see legend). (A) COI network based on 120 sequences, clustered in 29 haplotypes. (B) ZF network based on 174 sequences clustered in 21 haplotypes.

Analyses of the statistical parsimony networks (Fig. 3) revealed that the relative phylogenetic age of haplotypes is significantly related to both distribution range (Spearman's rank order correlation 0.46, *P* < 0.05 for COI and 0.46, *P* < 0.05 for ZF) and haplotype frequency (0.41, *P* < 0.05 for COI and 0.45, *P* < 0.05 for ZF) in both markers.

Log Bayes factor difference between the discrete and continuous model for COI was −669 and for ZF −215, thus indicating decisive support for the continuous model for both loci. The estimated age of the MRCA (i.e. the root of the tree) was 294 000 years (90–780 ka 95% HPD) for COI and 920 000 years (210–3600 ka) for ZF. The MRCAs of both loci originated most likely in Central Germany, however, with wide error margins (Figure S3).

Comparing the two continuous phylogeographic models with identical models except for an age constraint of 100 years for the root height (i.e. simulating a recent introduction with subsequent diversification) yielded in both cases decisive support for the unconstraint model (log Bayes factors for both COI and ZF > 100). The estimated mutation rates for the constraint model exceeded 30% per million years for both loci.

### Past demography

**Figure 4:**
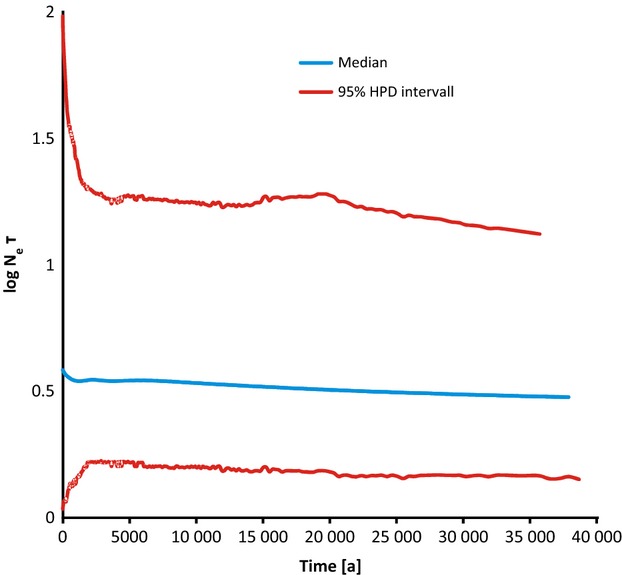
Extended Bayesian Skyline plot. The plot was derived from the combined cytochrome c oxidase subunit I and ZF alignments of Clade 1. The x axis is in units of years the y axis is equal to log Ne *τ* (the product of the effective population size and the generation length in years). The thick solid line is the median estimate, and the grey lines show the 95% HPD limits. The plot shows no indications of substantial effective population size dynamics for the last few 10 000 of years.

The extended Bayesian skyline analysis indicated little if any population growth during the last 35 000 years in the species, even considering the large 95% HPD intervals in particular towards the most recent past (Fig. 4). In the following analyses, we used therefore a constant size population model for simplicity. The analysis indicated a molecular clock rate of 0.0081/ma (0.015–0.002 95% HPD interval) for the ZF locus, assuming a rate of 0.05 ± 0.02 for the COI locus. Tajima's D was -0.98 (*P* > 0.10) for COI and −1.83 (*P* = 0.05) for ZF.

### Species distribution model, potential LGM refugia and genetic diversity

**Figure 5:**
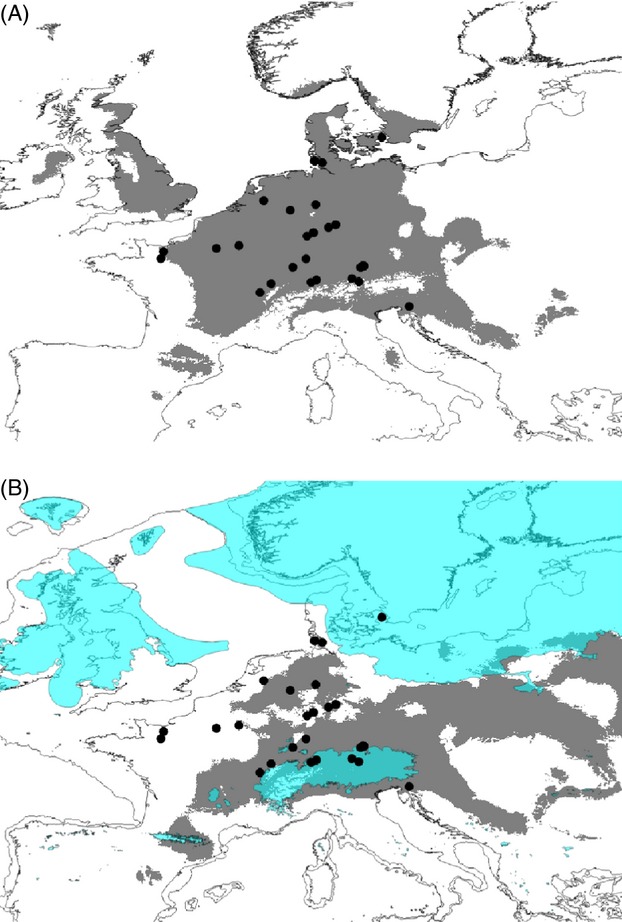
Species distribution modelling for Clade 1. The potential (A) present and (B) past (Last Glacial Maximum, LGM) distributions are shown in grey. Sites of genetically confirmed occurrence are shown as black circles. The figure (B) contains the LGM coastlines and the extent of the ice shield (petrol).

The species distribution modelling on the genetically confirmed points of presence did not include the presumed native areas with the exception of Southern England. Instead, it comprised parts of the Balkans, Northern Italy, Czech Republic, Slovakia and Poland (Fig. 5A). The estimated suitable species range during the LGM was quite large and covered large areas between the Scandinavian and the Alpine ice sheet (Fig. 5B). Among the 10 sites with the highest genetic diversity, nine were estimated as suitable during LGM for COI and five of 10 for ZF.

## Discussion

Our DNA-taxonomy approach indicated the dire need of a thorough integrative taxonomic revision of the entire genus *Arion*. The presence of many unnamed, mostly highly divergent haplotype clades calls for thorough integrative taxonomic studies on their specific status, as several previous studies in *Arion* have revealed the presence of undescribed species. (Pinceel et al. 2004; Pinceel 2005). However, they are not the focus of this contribution. Here, the more relevant finding is that all haplotype clades found have a more or less coherent geographic distribution, which is in most cases comparatively small, in particular in the south-west of the surveyed area. In general, no haplotype of a clade from Western France or Spain occurred in Central Europe or vice versa. A possible exception is a Spanish clade including a sequence from GenBank termed ‘*A. rufus’*, from which individuals also occurred in the United Kingdom. This suggests that there seems to be generally little propensity for passive dispersal and invasiveness in the genus (but see Pinceel et al. 2005b).

The *Arion* specimens that are perceived as invasive in Central Europe belong to a haplotype clade (Clade 1, Fig. 2) that has a geographically coherent distribution in Northern France, the Benelux states, Germany, Denmark and the Alpine arc (Fig. 1). We have not found a single individual of this clade in its generally presumed original range in Western France and north-west Spain. The Southern United Kingdom was admittedly not thoroughly sampled. However, this area was mentioned only by a single source on an unclear factual basis as potential area of origin and an origin there is not in line with the presumed invasion mechanism via agricultural goods. Moreover, even finding the species there would not compromise the below results. Given the necessarily incomplete sampling in terms of sites visited and samples sequenced, it is of course always possible that neither the exact area of origin for this particular clade was sampled nor that an individual of this clade was sampled even though it was there. Nevertheless, the sampling was thorough enough to suggest that the species, if present at all, cannot be very abundant and/or widespread in its purported native range. Both rarity and geographic restriction in the area of origin make a species not particularly prone to unintentional mass abduction (Kolar and Lodge 2001). An alternative explanation would be that it may have originated in Eastern Europe, the Balkans, Italy or elsewhere in an European area not or only superficially sampled, but the lack of evidence for the presence of the genetic lineage from the generally presumed area of origin already casted doubts on its invasive status. Because the presumed area of origin could not be confirmed, a comparison with the invasion area regarding the expected lack of evolutionary divergence (second prediction) was not possible.

Testing the third hypothesis concerning the expected population genetic structure of an invasive slug species yielded striking results. The finding that phylogenetically older haplotypes tended to be both more frequent and more widespread than younger ones (Fig. 4) indicated that the species is more or less in mutation–drift–migration equilibrium (Crandall and Templeton 1993). Both is to be expected in species that occupy their range for a time that allowed new haplotypes to arise locally by mutation and spread by distance-depended dispersal over millennia (Crandall and Templeton 1993), but not for a species introduced and spread only a few decades and thus few generations ago. Along this line of evidence argues also that the additional spatial information contained in the continuous phylogeographical distribution model, modelling the continuous phylogeographic diffusion through a continuous landscape, fitted the data much better than a discrete model, where dispersal among sampling sites is assumed to be independent (Lemey et al. 2010). The root for both loci was inferred to have been in Germany (Appendix S2), excluding, for example, the populations of Northern France as the source of a more or less recent expansion. The root localization, however, depends on the sampling design and may change with the inclusion of yet not sampled sites. To accommodate the spatial distribution pattern of genetic diversity found with the scenario of a recent introduction or expansion, we would have to assume molecular clock rates of more than 30% for the mitochondrial COI marker, respectively, 10% for the nuclear locus per one million years, which is far beyond any neutral rate ever proposed for the Metazoa (Baer et al. 2007).

Against expectation number four, we also found no indication of a demographic expansion. Even though Tajima's D, based on haplotype, frequency spectra were marginally significantly negative, indicating either a population expansion or purifying selection (Tajima 1989), the corresponding value of COI was not, which would be expected in case of an expansion, because demographic events affect all loci. On the contrary, the coalescence-based EBSP analysis (Fig. 4) indicated a particularly stable population size even during the postglacial warming, even though the most recent past was not clearly resolved by the approach. This is nonwithstanding the sometimes substantial short-term census population fluctuations in slugs, depending on ecological conditions (Godan 1979) which do not influence long-term estimates of effective population size, because the latter is determined by the average census size in bust rather than in boom times (Vucetich et al. 1997). However, due to the stochasticity of the coalescence process, the reliability of demographic inferences from molecular markers increases with their number (e.g. Hare 2001). Even though the congruence of the two markers used gives some confidence in the overall tendency, more markers would be needed to study the past demography in more detail.

Supporting evidence for an ancient Central European origin of the clade came from the climate niche modelling of the current distribution which shows that the presumed original range does not fall into the realised climatic niche. Projecting the current climate model on LGM condition additionally indicated that the presence of the species was possible in most of the present range during the LGM of the Pleistocene. This potential persistence in large parts of the present species range may also explain the lack of postglacial population expansion signal in the demographic analysis (Fig. 3). Additional evidence for this interpretation comes from the fact that most of the populations with the highest genetic diversity occurred within the inferred climatic LGM refugia (Cordellier and Pfenninger 2009). However, the climate niche estimate for the species depends on the sampling and may be modified by the inclusion of more confirmed occurrence points.

In conclusion, the population genetic structure of Clade 1, known as *A. lusitanicus* auct. non-Mabille or *A. vulgaris* showed neither the characteristics expected of an invasive species in general nor to the more specific expectations outlined previously. Studies of independently documented invasions of land snails yielded different results. In the invasive *Theba pisana,* the authors concluded from the absence of phylogeographic structure on repeated, intense human mediated dispersal except in the native range (Daumer et al. 2012). In a very thorough recent attempt to characterize the invasion dynamics of world-wide invasive populations of a helicid land snail *Cornu aspersum*, Guiller et al. (2012) found all predicted features of invasive populations they tested for: indication of a recent population expansion, lower nuclear and mitochondrial diversity in invasive populations, admixture from different sources in the invasion area. Instead in our case, all population genetic characteristics typically associated with a natural, ancient distribution were diagnosed. The inferred structure corresponded well to the increasing number of land snail species inferred to have survived the LGM in Northern refugia (Haase et al. 2003; Pfenninger et al. 2003; Pinceel et al. 2005a; Dépraz et al. 2008; Weigand et al. 2012).

We tried to gain corroborative evidence for the presence of Clade 1 in Central Europe prior to 1950 from various museum collections. Unfortunately, such samples either did not exist, or we were not able to obtain DNA of sufficient quality from them, or our requests to access such samples were not answered. However, DNA of sufficient quality for inclusion in our study can realistically only be expected from ethanol stored specimens of about 100 years of age (Wandeler, Hoeck, Keller 2007). But even finding *A. lusitanicus* auct. Non-Mabille individuals in samples from the first half of the twentieth century could not be regarded as decisive evidence for the proposed scenario as the anthropogenic introduction may well have started with an unnoticed lag phase a few decades earlier (Simberloff et al. 2013). Overall, there remained therefore little doubts that the tested populations of the focal species are native to Central Europe and not invasive. However, as we have investigated only this area, the situation might be different for England, where the species may be also native, Scandinavia (Proschwitz 1997) or Eastern Europe (Soroka et al. 2009).

After having established that the focal *Arion* species is most likely native in Central Europe, how did the perception of an invasive species gain ground in public and scientific opinion? We can only guess to suggest a plausible scenario. The taxonomic inventories, important field guides and keys of the first half of the twentieth century preferentially consulted by naturalists (Please find a list of some respective titles in Appendix S3) basically noted with few exceptions only two large arionid slugs of the species' size for Central Europe, in some cases even until the 1990s. (Over)simplifying the truly complex and disputed taxonomic history of the genus, these were, according to the taxonomic school followed, either from the *A. rufus/ater* L./*A. empiricorum* Férussac 1819 complex or from the *A. subfuscus* group, both with a presumed distribution over entire Europe. The important thing here is that in particular the field guides and keys noted the outward appearance of these species as highly variable in terms of colouration, size and body surface texture, thus well covering the cryptic species. So anybody going to the field and using the available literature was forced to the conclusion that the encountered specimens belong to one of these species. Even Godan (Godan 1979), recognizing the presence of *A. lusitanicus* as pest, mentions only *A. rufus* and *A. subfuscus* in the detailed lists of pest species and their impact, perhaps because these lists were compiled before the common recognition of *A. lusitanicus*. It was the merit of Schmid (1970) to provide a relatively easily accessible anatomical trait to distinguish the two. And with this means to distinguish the species, other malacologists became increasingly aware of the presence of a ‘new’ slug, necessarily on the expense of the perceived abundance and distribution of the previously synonymized species. Unfortunately, Schmid (1970) also followed erroneously the wrong attribution by authors across Europe of this and other taxa to the name *A. lusitanicus* Mabille 1868 (Castillejo 1997, 1998). And as *A. lusitanicus* was assumed to be a native from Portugal, the ‘logical’ conclusion was that this newly discovered species must be invasive. The sometimes enormous population size fluctuations of slugs (Godan 1979) may have in boom times additionally contributed to the public notion of a suddenly (over-)abundant slug of unknown origin. Obviously, nobody questioned the invasive status of the species even when it was unambiguously discovered that it is not the Portuguese *A. lusitanicus* Mabille 1868. The taxonomic status of the species remains thus uncertain.

Another, biologically more interesting question is whether our unnamed *Arion* species experienced a strong demographic increase during the time it was first noted and whether this contributed to the notion of an invasive species. Although it will be difficult to come *post hoc* to conclusive results, the changes of land use practice in terms of mechanisation and agrochemicals from the 1950s on, the *Flurbereinigung*, climate change, increasing reforestation and urbanisation may well have changed local distribution and abundance patterns in slug species. Increased interspecific interference competition, perhaps as a consequence of environmental changes, has been held responsible for changes in habitat use patterns in land snails (Kimura and Chiba 2010). Other biotic interactions can also play a role in changing abundance patterns. A recent study has shown that *A. lusitanicus* auct. Non-Mabille individuals mated at low rate under laboratory conditions with *A. rufus* (Dreijers et al. 2013). Even though no successful reproduction was observed in this study (Dreijers et al. 2013), the possibility of interspecific hybridisation cannot be excluded in sympatric populations. However, our data contained no indication of hybridisation such as divergent mitochondrial or nuclear haplotypes. It is also possible that evolutionary adaptation processes regarding behaviour or habitat preference made the species more conspicuous. Detailed population genomic analyses could help to shed more detailed light on recent evolutionary and demographic processes the species has undergone in the recent past.

We have shown that the invasion status of a species can be inferred based on *a priori* population genetic predictions even without knowledge of its ancestral population. This is particularly relevant for applied purposes, because management (e.g. Moss and Hermanutz 2010) or even eradication efforts crucially depend on whether a species is introduced or native (see respective paragraphs in, e.g., the Convention on Biological Diversity or the Convention on Migratory Species of Wild Animals). Moreover, the species is used to test predictions on invasive species success, for example, regarding phenotypic plasticity (Knop and Reusser 2012) and diet choice (Zaller et al. 2013) or as an example of their impact (e.g. Blattmann et al. 2013), which is questionable given the presented results. We thus argue that our approach should be applied as cautionary measure in cases where the *a priori* evidence for anthropogenic introduction is poor or nonexistent before attributing the status of invasiveness with the above-mentioned consequences for biodiversity management and applied research.

## Data archiving statement

Data for this study are available at: GenBank.

## Supporting Information

**Appendix S1.** Development of ZF locus. **Table S1.** Primer for the amplification of COI, ZF and ND1. **Table S2.** Concentrations of chemicals for PCRs (total volume 25 μL). **Table S3.** Protocols for PCRs. **Appendix S2.** **Figure S1.** ML phylogeny of ND1 locus. **Figure S2.** Distribution of clades sampled in the study. **Figure S3.** Estimated 80% HDP of location of origin of COI haplotypes MRCA (white shade) and ZF haplotypes (red shade) for Clade 1. **Table S1.** Sampling sites and clade distribution. List of 166 GenBank Accession Numbers with attributed species for COI used for comparative purposes. List of 40 ND1 Accession Numbers used. **Appendix S3.** List of taxonomic treatises, determination keys, field guides, etc. consulted.
