# Supplementary material for: Misperceived invasion: the Lusitanian slug (Arion lusitanicus auct. non-Mabille or Arion vulgaris Moquin-Tandon 1855) is native to Central Europe
Source: Evol Appl. 2014 Jun 17;7(6):702–13. doi: 10.1111/eva.12177 (PMC4105919; doi:10.1111/eva.12177)
Supplement: Supplementary file 1 [file eva0007-0702-SD1.pdf]

Supplementary Information 1

Development of ZF locus

For individuals of the clade identified as belonging to the focal species, we sequenced additionally a nuclear locus. Primers for this locus were developed from a low coverage (<10%) genomic shotgun on a 454 platform (for a principle account on the proceeding see Feldmeyer et al. 2010). Briefly, we x-blasted singleton reads larger than 500 bp against the non-redundant protein data base of NCBI and retained six reads with e-values below e-5. For these reads, we developed primers with the online tool Primer3 (Rozen and Skaletsky, 1998; http://biotools.umassmed.edu/bio apps/primer3_www.cgi). Of the six reads, four yielded PCR products of the expected length. Sequencing the products as described above, a single locus, with a significant BLAST hit against a zinc-finger protein, appeared to amplify a single locus and proved to be polymorphic.

Table 1. Primer for the amplification of COI, ZF and ND1

Primer	5’-3’ Sequence	Reference
LCOI (forward)	GGTCAACAAATCATAAAGATATTGG	Folmer et al. 1994
HCOI (reverse)	TAAACTTCAGGGTGACCAAAAAATCA	
ZF (forward)	TGTTTGGAATCTAGAGCCTGA	results of own shot gun sequencing
ZF (reverse)	TTTGTTGCTGTCCTGCTTGT	
MOL_NAD1F (forward)	CGRAARGGMCCTAACAARGTTGG	Quinteiro et al. 2005
MOL_NAD1R (reverse)	GGRGCACGATTWTGCTCNGCA	

Table2. Concentrations of chemicals for PCRs (total volume 25µl)

Locus	Chemical	Volume (µl)
COI and ZF	10x buffer	2.5
	100 mM M_gCl_2	2.0
	20 mM dNTP-Mix	0.3
	10 pmol LCOI	1.0
	10 pmol HCOI	1.0
	Taq-Polymerase	0.3
	ddH_2O	11.4
	Bovine Serum Albumin (10 mg/mL)	1.5
	Template DNA	5.0
ND1	10x buffer	2.5
	100 mM M_gCl_2	2.0
	20 mM dNTP-Mix	0.3
	10 pmol LCOI	1.0
	10 pmol HCOI	1.0
	Taq-Polymerase	0.3
	ddH_2O	11.15
	Bovine Serum Albumin (10 mg/mL)	1.5
	Dimethylsulfoxid (DMSO)	0.25
	Template DNA	5.0

Table 3. Protocols for PCR reactions

COI:

Step	Temperature (°C)	Time (min.)	Iterations
Denaturation	95	01:00	
Denaturation	95	00:30	30
Annealing	52 / 45	00:30	30
Elongation	72	00:30	30
Final elongation	72	03:00	
Storage	8	∞	∞

ZF and ND1:

Step	Temperature (°C)	Time (min.)	Iterations
Denaturation	95	01:00	
Denaturation	95	00:30	30
Annealing	52	00:30	30
Elongation	72	00:30	30
Final elongation	72	03:00	
Storage	8	∞	∞

Supplementary Information 2

Figure 1. ML phylogeny of ND1 locus. The topotypic *A. lusitanicus* does not cluster with the *A. lusitanicus* from the invasion area. The ND1 locus does not resolve *A. rufus* from the latter. Numbers on nodes present bootstraps > 90%.

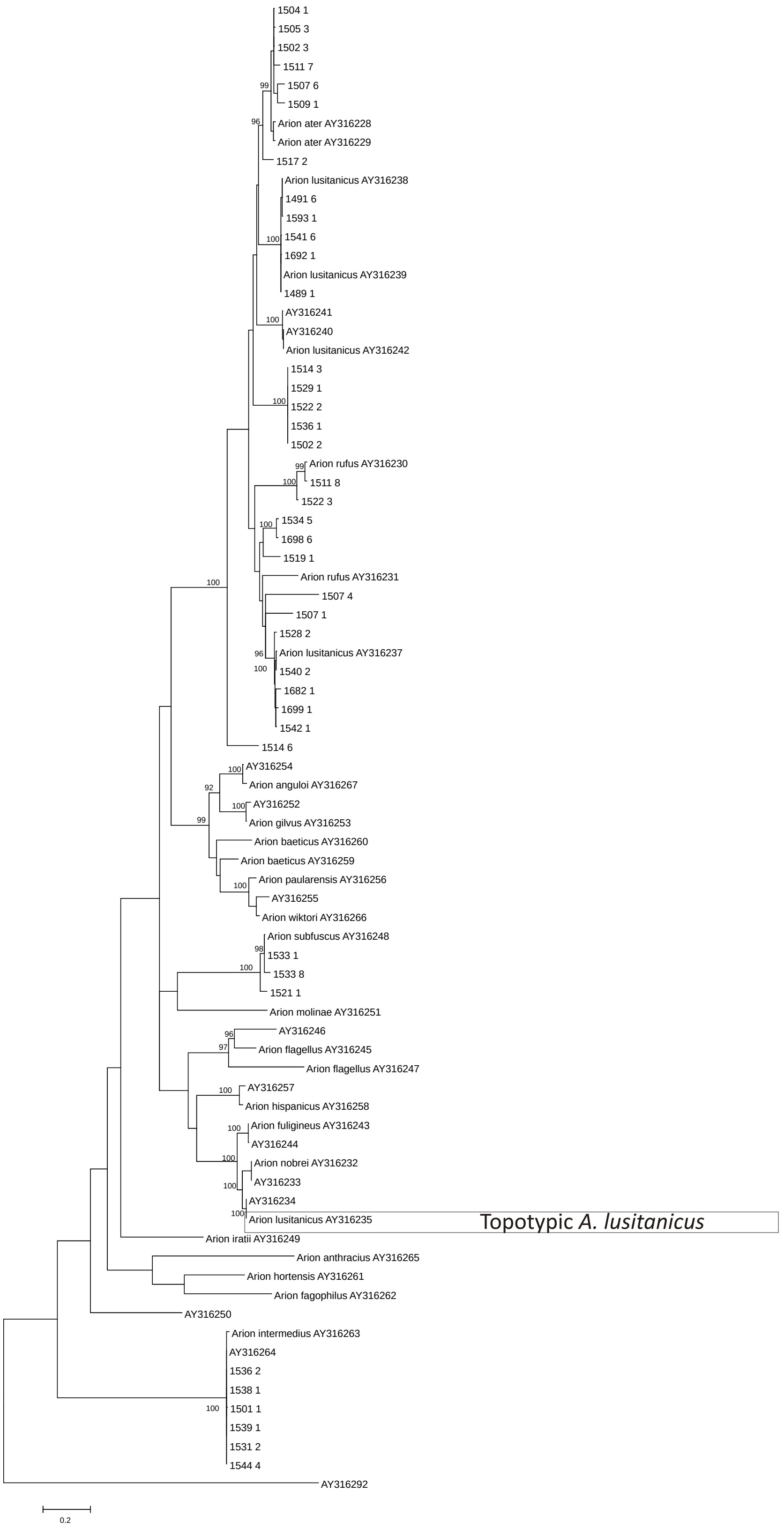

Figure 2. Distribution of clades sampled in the study. The numbering follows Figure 2 of the article.

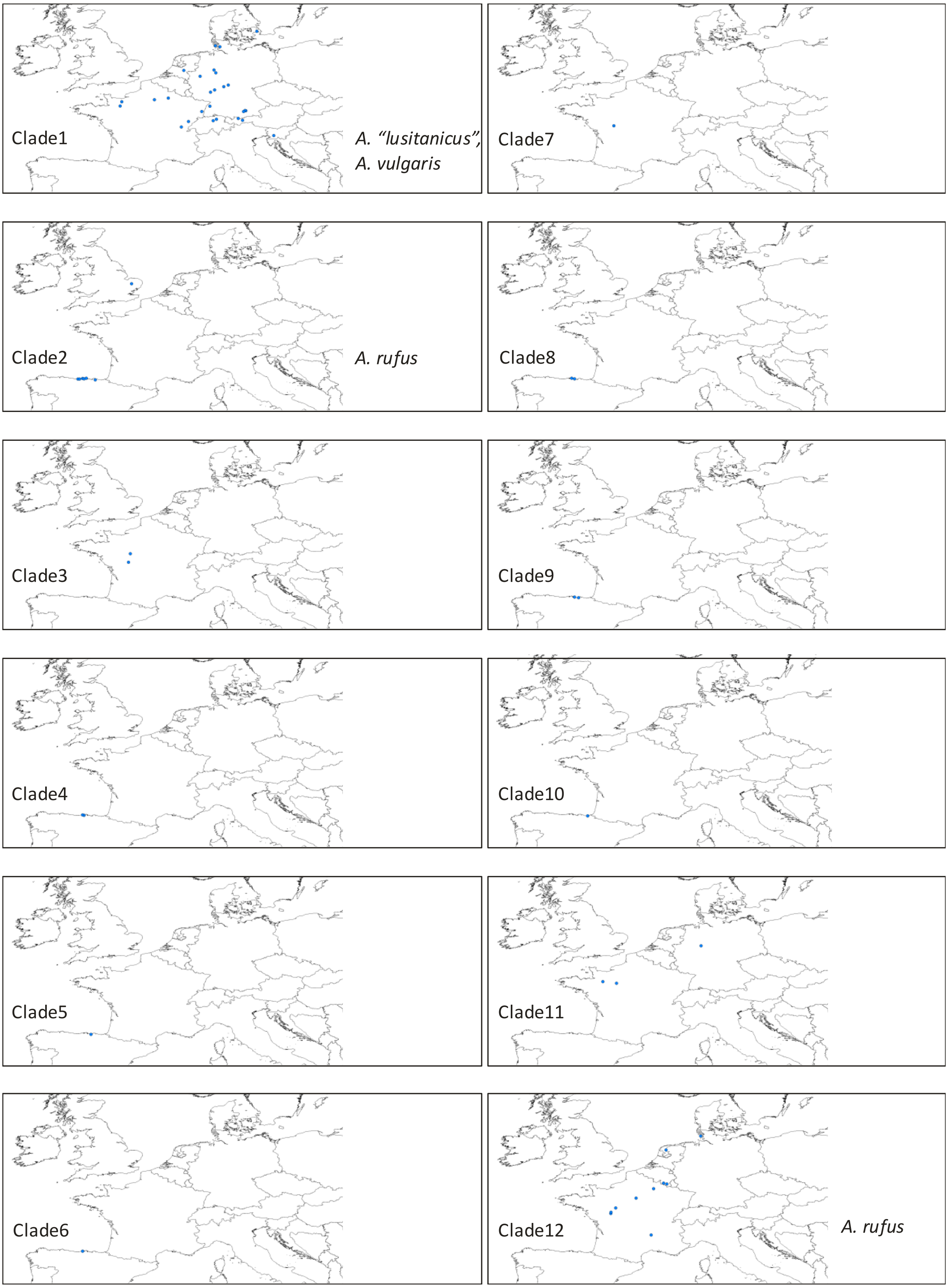

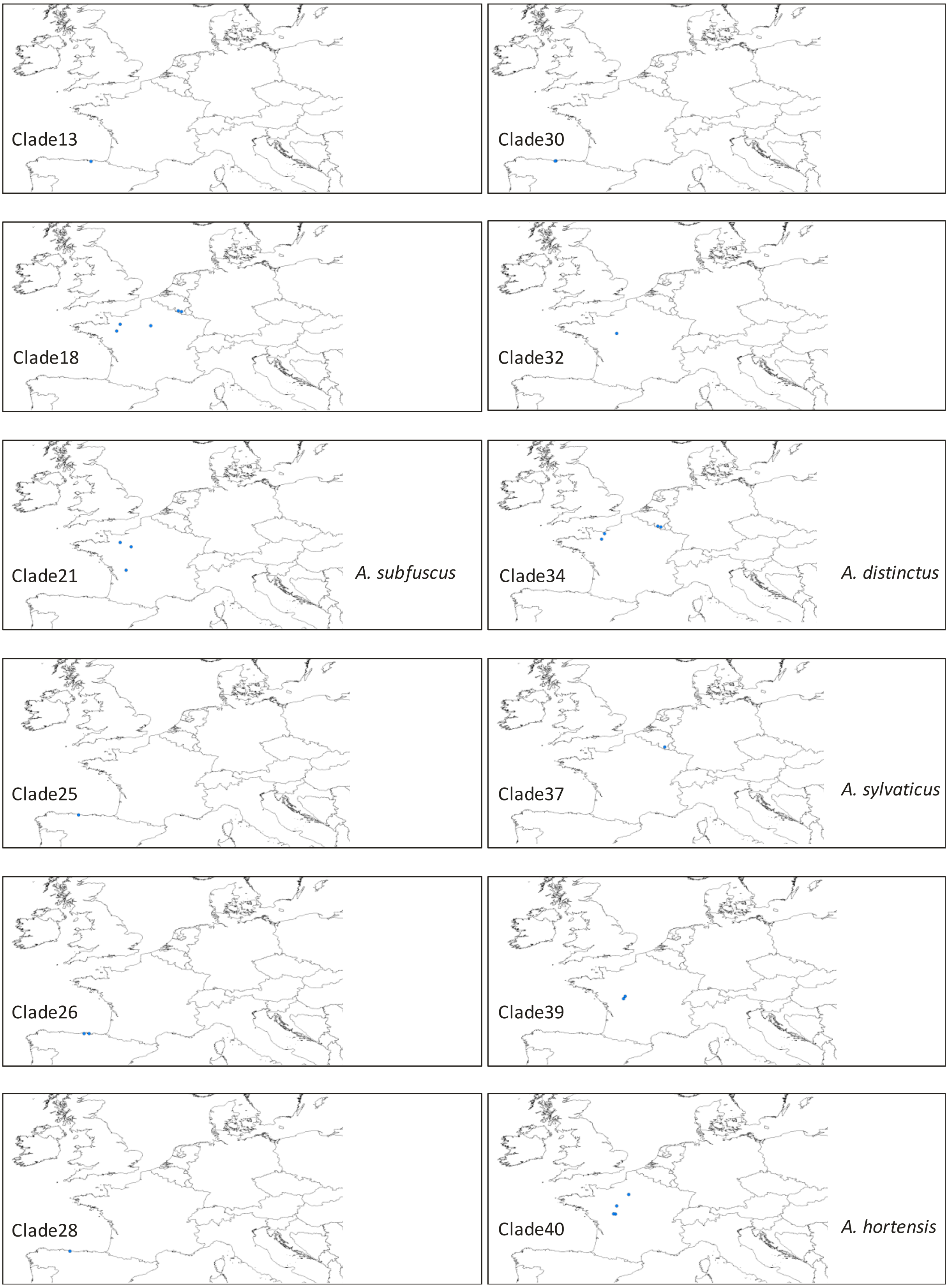
Clade13
Clade30
Clade18
Clade32
Clade21
A. subfuscus
Clade34
A. distinctus
Clade25
Clade37
A. sylvaticus
Clade26
Clade39
Clade28
Clade40
A. hortensis

Figure 3. Estimated 80% HDP of location of origin of COI haplotypes MRCA (white shade) and ZF haplotypes (red shade) for Clade 1. The estimated age of the MRCA is 294,000 years (90-780 ka 95% HPD) for COI and 920,000 years (210-3600 ka) for ZF.

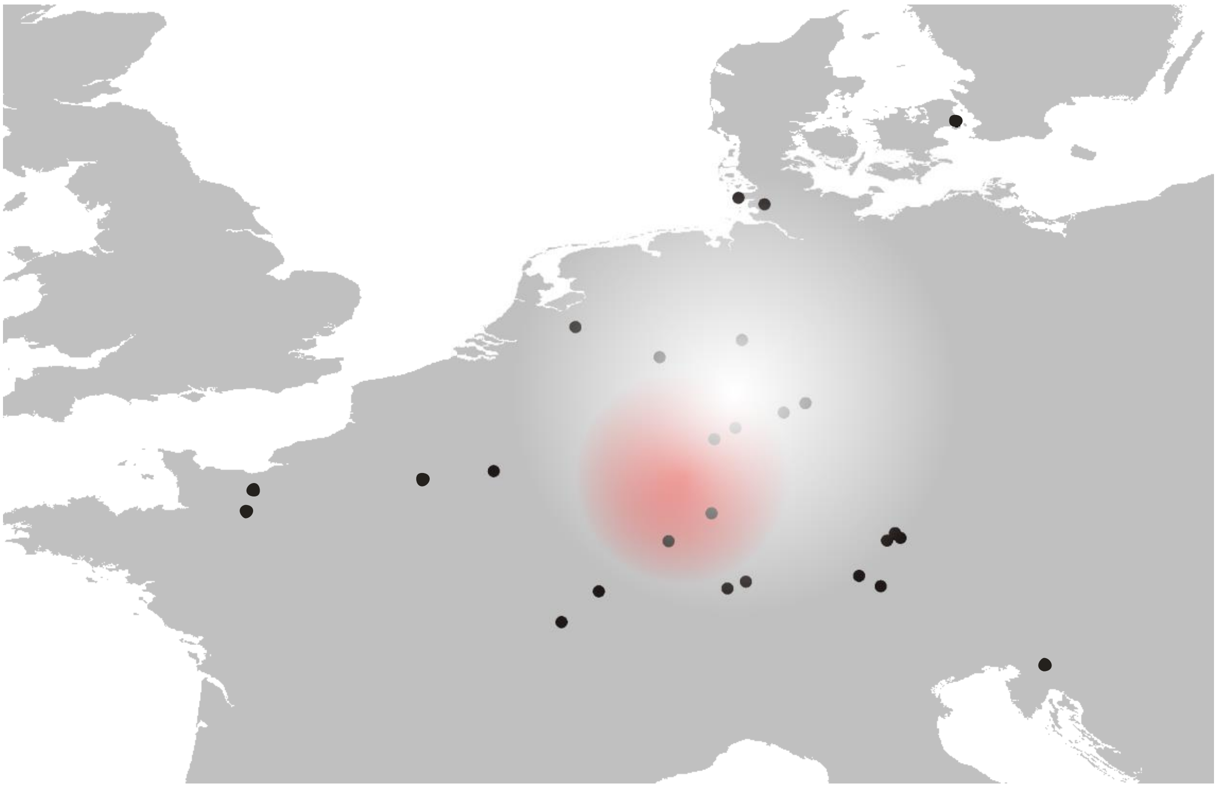

Table 1. Sampling sites and clade distribution.

No	Site	Country	Location	Latitude	Longitude	Number individuals sequenced COI	Number individuals sequenced ND1	Number individuals sequenced ZF	Clade 1	2	3	4	5	6	7	8	9	10	11	12	13	18	21	25	26	28	30	32
1	1489	France	Colmar	48.068	7.380	9	1	8	9	-	-	-	-	-	-	-	-	-	-	-	-	-	-	-	-	-	-	-
2	1491	France	Ornans	47.109	6.113	4	1	2	4	-	-	-	-	-	-	-	-	-	-	-	-	-	-	-	-	-	-	-
3	1492	France	Vercia	46.591	5.452	7	-	6	7	-	-	-	-	-	-	-	-	-	-	-	-	-	-	-	-	-	-	-
4	1494	France	Chateau de l'Herm	44.963	3.976	6	-	-	-	-	-	-	-	-	-	-	-	-	-	6	-	-	-	-	-	-	-	-
5	1498	Spain	Ribadesella	43.450	-5.121	9	-	-	-	-	-	-	-	-	-	-	-	-	-	-	-	-	-	6	-	3	-	-
6	1501	Spain	Serdio	43.368	-4.444	4	1	-	-	-	-	-	-	-	-	-	-	-	-	-	-	-	-	-	-	-	4	-
7	1502	Spain	La Brana	43.388	-4.373	2	2	-	-	1	-	-	-	-	-	-	-	-	-	-	-	-	-	-	-	-	1	-
8	1504	Spain	Alfoz de Lloredo	43.387	-4.206	4	2	-	-	4	-	-	-	-	-	-	-	-	-	-	-	-	-	-	-	-	-	-
9	1507	Spain	Piélagos	43.441	-3.924	5	3	-	-	1	-	2	-	2	-	-	-	-	-	-	-	-	-	-	-	-	-	-
10	1509	Spain	Marina de Cudeyo	43.410	-3.794	7	1	-	-	1	-	5	-	-	-	-	-	-	-	-	-	-	-	-	1	-	-	-
11	1511	Spain	Arnuero	43.470	-3.572	6	2	-	-	1	-	-	-	-	-	5	-	-	-	-	-	-	-	-	-	-	-	-
12	1512	Spain	Castro Urdiales	43.403	-3.304	3	-	-	-	-	-	-	-	-	-	1	1	-	-	-	-	-	-	-	1	-	-	-
13	1514	Spain	Muskiz	43.333	-3.134	4	2	-	-	-	-	-	3	-	-	-	-	-	-	-	1	-	-	-	-	-	-	-
14	1516	Spain	Laukiz	43.345	-2.913	1	-	-	-	-	-	-	-	-	-	-	1	-	-	-	-	-	-	-	-	-	-	-
15	1517	Spain	Eleizalde-Olabarri	43.315	-2.718	4	1	-	-	4	-	-	-	-	-	-	-	-	-	-	-	-	-	-	-	-	-	-
16	1519	Spain	Usúrbil	43.279	-2.084	5	1	-	-	-	-	-	-	-	-	-	-	5	-	-	-	-	-	-	-	-	-	-
17	1521	France	Verteuil-sur-Charente	45.957	0.202	4	1	-	-	-	-	-	-	-	-	-	-	-	-	-	-	-	4	-	-	-	-	-
18	1522	France	Dissay	46.705	0.433	6	2	-	-	-	1	-	-	-	3	-	-	-	-	-	-	-	-	-	-	-	-	-
19	1524	France	Dange St. Romain	46.935	0.592	6	-	-	-	-	-	-	-	-	-	-	-	-	-	-	-	-	-	-	-	-	-	-
20	1525	France	Richelieu	46.957	0.403	9	-	-	-	-	-	-	-	-	-	-	-	-	-	-	-	-	-	-	-	-	-	-
21	1526	France	Loudon	47.009	0.135	4	-	-	-	-	-	-	-	-	-	-	-	-	-	4	-	-	-	-	-	-	-	-
22	1528	France	Seuilly	47.124	0.164	6	1	-	-	-	-	-	-	-	-	-	-	-	-	6	-	-	-	-	-	-	-	-
23	1529	France	Semblança	47.522	0.611	5	1	-	-	-	2	-	-	-	-	-	-	-	-	2	-	-	-	-	-	-	-	-
24	1531	France	La Cuverie	47.708	0.709	4	1	-	-	-	-	-	-	-	-	-	-	-	-	-	-	-	-	-	-	-	-	1
25	1532	France	Origné	47.944	-0.697	10	-	1	-	-	-	-	-	-	-	-	-	-	-	-	-	1-	-	-	-	-	-	-
26	1533	France	Commer	48.173	0.680	8	2	-	-	-	-	-	-	-	-	-	-	-	1	-	-	-	7	-	-	-	-	-
27	1534	France	La Haie-Traversaine	48.335	-0.617	6	1	-	-	-	-	-	-	-	-	-	-	-	6	-	-	-	-	-	-	-	-	-

28	1535	France	La Ferte-Macé	48.579	-0.368	9	2	-	2	-	-	-	-	-	-	-	-	-	-	-	-	1	3	-	-	-	-	-
29	1537	France	Pontigny	48.997	-0.204	2	-	2	2	-	-	-	-	-	-	-	-	-	-	-	-	-	-	-	-	-	-	-
30	1538	France	La Houblonniere	49.120	-0.069	3	1	-	-	-	-	-	-	-	-	-	-	-	-	-	-	-	-	-	-	-	-	-
31	1539	France	Galluis	48.802	1.850	2	1	-	-	-	-	-	-	-	-	-	-	-	-	-	-	-	-	-	-	-	-	-
32	1540	France	Fleury en Bière	48.455	2.550	2	1	-	-	-	-	-	-	-	-	-	-	-	-	1	-	1	-	-	-	-	-	-
33	1541	France	Boissy-Fressnoy	49.182	2.891	6	1	4	6	-	-	-	-	-	-	-	-	-	-	-	-	-	-	-	-	-	-	-
34	1542	France	Wameriville	49.348	4.213	3	1	1	1	-	-	-	-	-	-	-	-	-	-	1	-	-	-	-	-	-	-	-
35	1544	Belgium	Fays-les-Veneurs	49.865	5.161	3	1	-	-	-	-	-	-	-	-	-	-	-	-	1	-	1	-	-	-	-	-	-
36	1545	Belgium	Assenois	49.797	5.456	10	-	-	-	-	-	-	-	-	-	-	-	-	-	2	-	2	-	-	-	-	-	-
37	1548	Switzerland	Aathal	47.334	8.762	3	-	2	3	-	-	-	-	-	-	-	-	-	-	-	-	-	-	-	-	-	-	-
38	1593	Germany	München	48.145	11.544	6	1	6	6	-	-	-	-	-	-	-	-	-	-	-	-	-	-	-	-	-	-	-
39	1594	Germany	München	48.142	11.564	4	-	4	4	-	-	-	-	-	-	-	-	-	-	-	-	-	-	-	-	-	-	-
40	1595	Germany	München	48.164	11.482	6	-	5	6	-	-	-	-	-	-	-	-	-	-	-	-	-	-	-	-	-	-	-
41	1653	Slovenia	Postojna	45.777	14.211	2	-	2	2	-	-	-	-	-	-	-	-	-	-	-	-	-	-	-	-	-	-	-
42	1678	Germany	Ottenhöfen	48.567	8.157	8	-	6	8	-	-	-	-	-	-	-	-	-	-	-	-	-	-	-	-	-	-	-
43	1679	Germany	Hattingen	51.407	7.230	2	-	1	2	-	-	-	-	-	-	-	-	-	-	-	-	-	-	-	-	-	-	-
44	1682	Niederlande	Makkum	53.005	5.379	5	1	-	-	-	-	-	-	-	-	-	-	-	-	5	-	-	-	-	-	-	-	-
45	1683	Germany	Heide	54.212	9.108	9	-	1	9	-	-	-	-	-	-	-	-	-	-	-	-	-	-	-	-	-	-	-
46	1684	Germany	Hintersteinau	50.419	9.470	3	-	3	3	-	-	-	-	-	-	-	-	-	-	-	-	-	-	-	-	-	-	-
47	1685	Österreich	Sellrain	47.233	11.244	2	-	2	2	-	-	-	-	-	-	-	-	-	-	-	-	-	-	-	-	-	-	-
48	1686	Germany	Nieder-Olm	49.907	8.214	2	-	2	2	-	-	-	-	-	-	-	-	-	-	-	-	-	-	-	-	-	-	-
49	1687	Germany	St. Peter-Ording 1	54.306	8.663	5	-	-	5	-	-	-	-	-	-	-	-	-	-	-	-	-	-	-	-	-	-	-
50	1689	England	Thetford Forest	52.431	0.723	1	-	-	-	1	-	-	-	-	-	-	-	-	-	-	-	-	-	-	-	-	-	-
51	1690	Danmark	Kopenhagen	55.692	12.598	3	-	1	3	-	-	-	-	-	-	-	-	-	-	-	-	-	-	-	-	-	-	-
52	1691	Netherlands	Wageningen	51.977	5.675	3	-	2	3	-	-	-	-	-	-	-	-	-	-	-	-	-	-	-	-	-	-	-
53	1692	Germany	Frankfurt	50.111	8.590	2	1	1	2	-	-	-	-	-	-	-	-	-	-	-	-	-	-	-	-	-	-	-
54	1693	Austria	Bichlbach	47.417	10.842	2	-	2	2	-	-	-	-	-	-	-	-	-	-	-	-	-	-	-	-	-	-	-
55	1694	Germany	Elters	50.580	9.890	5	-	4	5	-	-	-	-	-	-	-	-	-	-	-	-	-	-	-	-	-	-	-
56	1695	Germany	Bielefeld	52.007	8.532	1	-	1	1	-	-	-	-	-	-	-	-	-	-	-	-	-	-	-	-	-	-	-
57	1696	Germany	Giesing	48.062	11.351	7	-	7	7	-	-	-	-	-	-	-	-	-	-	-	-	-	-	-	-	-	-	-
58	1697	Switzerland	Aathal	47.200	8.457	3	-	3	3	-	-	-	-	-	-	-	-	-	-	-	-	-	-	-	-	-	-	-

59	1698	Germany	Paderborn	51.741	8.715	7	1	6	6	-	-	-	-	-	-	-	-	-	1	-	-	-	-	-	-	-	-	-
60	1699	Germany	St. Peter-Ording 2	54.317	8.666	6	1	2	5	-	-	-	-	-	-	-	-	-	-	1	-	-	-	-	-	-	-	-
∑						285	39	87	120	13	3	7	3	2	3	6	2	5	8	29	1	5	14	6	2	3	5	

1

List of 166 GenBank Accession nos. with attributed species for COI used for comparative purposes.

EF520640 Arion lusitanicus
EF520641 Arion lusitanicus
EF520642 Arion lusitanicus
EF520643 Arion lusitanicus
EU734823 Arion lusitanicus
EU734824 Arion lusitanicus
EU734825 Arion lusitanicus
EU734826 Arion lusitanicus
GQ166169 Arion lusitanicus
AY987903 Arion rufus
AY987902 Arion rufus
EF520647 Arion rufus
FJ481178 Arion rufus
AY987901 Arion rufus
EF520644 Arion rufus
AY987900 Arion rufus
EF520646 Arion rufus
EF520645 Arion rufus
AY987874 Arion distinctus
AY987875 Arion distinctus
AY423698 Arion distinctus
AY423700 Arion distinctus
EF128218 Arion distinctus
AY423697 Arion distinctus
AY423694 Arion distinctus
AY423692 Arion distinctus
AY423696 Arion distinctus
AY423701 Arion distinctus
AY423699 Arion distinctus
AY987879 Arion fasciatus
AY987877 Arion fasciatus
AY987878 Arion fasciatus
AY987880 Arion flagellus
AY987881 Arion flagellus
AY987882 Arion flagellus
AY987883 Arion franciscoloi
AY987884 Arion franciscoloi
AJ809429 Arion fuscus
AJ809414 Arion fuscus
AJ809423 Arion fuscus
AJ809421 Arion fuscus
AJ809434 Arion fuscus
AJ809416 Arion fuscus
AJ809410 Arion fuscus
AJ809409 Arion fuscus
AJ809430 Arion fuscus
AJ809445 Arion fuscus
AJ809408 Arion fuscus
AY987885 Arion fuscus
AJ809415 Arion fuscus
AJ809442 Arion fuscus
AJ809443 Arion fuscus
AJ809437 Arion fuscus
AY987887 Arion fuscus
AJ809422 Arion fuscus
AJ809413 Arion fuscus
AJ809440 Arion fuscus
AY987886 Arion fuscus
AJ809411 Arion fuscus
AJ809420 Arion fuscus
AJ809436 Arion fuscus
AJ809432 Arion fuscus
AJ809444 Arion fuscus
AJ809439 Arion fuscus
AJ809412 Arion fuscus
AJ809427 Arion fuscus
AJ809433 Arion fuscus
AJ809417 Arion fuscus
AJ809425 Arion fuscus
AJ809441 Arion fuscus
AJ809426 Arion fuscus
AJ809418 Arion fuscus
AJ809431 Arion fuscus
AJ809435 Arion fuscus
AJ809419 Arion fuscus
AJ809446 Arion fuscus
AJ809424 Arion fuscus
AJ809428 Arion fuscus
AJ809438 Arion fuscus
EU382742 Arion hortensis
AY423670 Arion hortensis
AY423671 Arion hortensis
AY423680 Arion hortensis
AY423681 Arion hortensis
AY423673 Arion hortensis
AY423687 Arion hortensis
AY423678 Arion hortensis
AY423679 Arion hortensis
AY423675 Arion hortensis
AY423674 Arion hortensis
AY423688 Arion hortensis
AY423683 Arion hortensis
AY423682 Arion hortensis
AY423677 Arion hortensis
AY423685 Arion hortensis
AY423691 Arion hortensis
AY423684 Arion hortensis
AY423672 Arion hortensis
AY423690 Arion hortensis
AY423676 Arion hortensis
AY987889 Arion hortensis
AY987888 Arion hortensis
AY423686 Arion hortensis
AY423689 Arion hortensis
AY987891 Arion intermedius
EU382755 Arion intermedius
EU382756 Arion intermedius
AY987890 Arion intermedius
EU382748 Arion intermedius
EU382746 Arion intermedius
EU382751 Arion intermedius
EU382744 Arion intermedius
EU382753 Arion intermedius
EU382750 Arion intermedius
AY423705 Arion intermedius
EU382745 Arion intermedius
EU382752 Arion intermedius
EU382754 Arion intermedius
EU382749 Arion intermedius
EU382743 Arion intermedius
EU382747 Arion intermedius
AY987892 Arion iratii
AY987893 Arion lizarrustii
AY987896 Arion molinae
FJ348255 Arion occultus
FJ348254 Arion occultus
AY423702 Arion owenii
AY423704 Arion owenii
AY423703 Arion owenii
AY987897 Arion owenii
AY987898 Arion owenii
AY987899 Arion paularensis
AF513018 Arion silvaticus
AY987917 Arion silvaticus
AY987918 Arion silvaticus
AY987916 Arion subfuscus
AY987909 Arion subfuscus
AY987911 Arion subfuscus
GU249577 Arion subfuscus
GU249582 Arion subfuscus
GU249580 Arion subfuscus
GU249578 Arion subfuscus
AY987904 Arion subfuscus
GU249579 Arion subfuscus
AY987914 Arion subfuscus
GU249583 Arion subfuscus
GU249581 Arion subfuscus
GU249584 Arion subfuscus
GU249586 Arion subfuscus
AY987912 Arion subfuscus
AY987906 Arion subfuscus
AY987905 Arion subfuscus
AY987907 Arion subfuscus
AY987908 Arion subfuscus
AY987910 Arion subfuscus
GU249576 Arion subfuscus
GU249587 Arion subfuscus
AY987913 Arion subfuscus
GU249585 Arion subfuscus
AY987915 Arion subfuscus
AY943858 Arion transsylvanus
AY943860 Arion transsylvanus
AY943859 Arion transsylvanus
AY987919 Arion urbiae
AY987921 Arion wiktori
AY987920 Arion wiktori

List of 40 ND1 accession nos. used.

AY316292
AY316261
AY316262
AY316250
AY316265
AY316249
AY316263
AY316264
AY316251
AY316247
AY316246
AY316245
AY316254
AY316267
AY316252
AY316253
AY316256
AY316255
AY316266
AY316260
AY316259
AY316257
AY316258
AY316243
AY316244
AY316232
AY316233
AY316234
AY316235
AY316248
AY316230
AY316238
AY316239
AY316240
AY316242
AY316241
AY316228
AY316229
AY316231
AY316237

Supplementary Information 3

List of taxonomic treatises, determination keys, field guides, etc. consulted.

- Geyer D (1909) Die Weichtiere Deutschlands. Strecker & Schröder, Stuttgart.
- Lehmann FX (1884) Einführung in die Molluskenfauna des Grossherzigtums Baden. G. Braun’sche Hofbuchhandlung, Karlsruhe.
- Clessin (1884) Deutsche Excursions-Mollusken-Fauna. 2. Auflage, Bauer & Raspe, Nürnberg.
- Wiktor A (1973) Die Nacktschnecken Polens. Wydawnictwo Navkowe, Warszawa.
- Ehrmann P (1937) Mollusca. In: Die Tierwelt Mitteleuropas Band II. (Eds: Brohmer P, Ehrmann P, Ulmer G). Quelle & Meyer, Leipzig.
- Adam (1947) Revision des Mollusques des la Belgique I: Mollusques terrestres et dulcicoles. Memoires du Musée Royal d’Histoire Naturelle de Belgique No. 106, Brussels.
- Germain (1931) Mollusques terrestes et fluviatiles. Faune de la France tome 22. Librairie de la Faculté des Sciences.
- Lehmann A (1904) Die Schnecken und Muscheln Deutschlands. Förster & Borries, Zwickau.
- Hesse P (1926) Die Nacktschnecken der palaearktischen Region. Abhandlungen des Archiv für Molluskenkunde 2. Verlag der Malakologischen Gesellschaft, Frankfurt am Main.
- Schaefer M (1992) Brohmer: Fauna von Deutschland. 18. Auflage, Quelle & Meyer Verlag, Heidelberg.
- Stresemann E (1976) Exkursionsfauna für die Gebiete der DDR und der BRD - Wirbellose 1. Volk und Wissen Volkseigener Verlag, Berlin.
